# Detection of Enterotoxigenic *Escherichia coli* and Clostridia in the Aetiology of Neonatal Piglet Diarrhoea: Important Factors for Their Prevention

**DOI:** 10.3390/life13051092

**Published:** 2023-04-27

**Authors:** Nikolaos Tsekouras, Eleftherios Meletis, Polychronis Kostoulas, Georgia Labronikou, Zoi Athanasakopoulou, Georgios Christodoulopoulos, Charalambos Billinis, Vasileios G. Papatsiros

**Affiliations:** 1 Clinic of Medicine, Faculty of Veterinary Science, University of Thessaly, 43100 Karditsa, Greece; 2 Faculty of Public and Integrated Health, University of Thessaly, 43100 Karditsa, Greece; 3 Swine Technical Support, Hipra Hellas SA, 10441 Athens, Greece; 4 Department of Microbiology and Parasitology, Faculty of Veterinary Science, University of Thessaly, 43100 Karditsa, Greece; 5 Department of Animal Science, Agricultural University of Athens, 75 Iera Odos Street, Botanikos, 11855 Athens, Greece

**Keywords:** *E. coli*, ETEC, *C. difficile*, neonatal diarrhoea, piglets

## Abstract

This study aimed to research the involvement of enterotoxigenic *E. coli* (ETEC) and *C. difficile* or *C. perfringens* type C in the aetiology of neonatal piglet diarrhoea in Greece and to identify preventive factors for them. A total of 78 pooled faecal samples were collected randomly from 234 suckling piglets (1–4 days of age) with diarrhoea from 26 pig farms (3 piglets × 3 litters × 26 farms = 234 piglets = 78 faecal pool samples). The collected samples were initially screened for the presence of *E. coli* and *C. difficile* or *C. perfringens* via cultivation on MacConkey and anaerobic blood agar, respectively. Subsequently, the samples were pooled on ELUTE cards. From samples tested, 69.23% of those in the farms were ETEC F4-positive, 30.77% were ETEC F5-positive, 61.54% ETEC were F6-positive, 42.31% were ETEC F4- and *E. coli* enterotoxin LT-positive, 19.23% were ETEC F5- and LT-positive, 42.31% were ETEC F6- and LT-positive, while LT was found in 57.69% of those in the farms. *C. difficile* was involved in many cases and identified as an emerging neonatal diarrhoea etiological agent. Specifically, Toxin A of *C. difficile* was found in 84.62% and Toxin B in 88.46% of those in the farms. Antibiotic administration to sows in combination with probiotics or acidifiers was revealed to reduce the detection of antigens of ETEC and the enterotoxin LT of *E. coli*.

## 1. Introduction

Neonatal piglet diarrhoea is a major economic and welfare issue in the swine industry worldwide due to increased pre-weaning mortality and therapeutic cost, as well as decreased growth rates in piglets [1,2]. To this day, it has proven to be challenging to identify the agents responsible for neonatal diarrhoea clinical signs in pig herds [3]. Several previous studies have reported various common pathogens as etiological agents, including *Escherichia coli* (*E. coli*), *Clostridium perfringens* (*C. perfringens*), *Clostridioides difficile* (*C. difficile*), *Cystoisospora suis*, rotavirus, and *Cryptosporidium parvum* [4,5,6,7,8]. In many field cases, combinations of pathogens have been isolated [8]. 

Neonatal diarrhoea is one of the most frequent clinical signs in newborn piglets, significantly increasing pre-weaning mortality and the number of weaning piglets with a lower body weight than the farm’s target [9]. Even though enteric diseases in newborn piglets are often endemic, outbreaks characterized by high morbidity and mortality have been reported [10,11]. For example, in Sweden and Denmark, diarrhoea represented 5–24% of the overall pre-weaning mortality cases [12] and is responsible for a reduction in average daily gain (8–14 g/day) during the first week of life [13,14]. The cost of neonatal diarrhoea in affected pig herds was recently estimated to be EUR 134 per sow per year [2]. Commercial pig farms use vaccinations against *E. coli* and clostridial diseases, antibiotics, and alternatives to antibiotics to prevent or reduce economic losses due to neonatal diarrhoea. 

*E. coli* is a Gram-negative bacterium that normally lives in the intestine, but imbalances in the gut microbiota can potentially cause diarrhoea in pigs [15]. Neonatal piglets affected by *E. coli* suffer from severe diarrhoea and have a high mortality rate [16]. Enteropathogenic *E. coli* strains (EPEC) and enterotoxigenic *E. coli* strains (ETEC) are classified by their virulence factors, with EPEC being characterized by the production of intimin and ETEC strains by their principal virulence factors, enterotoxins (STa, STb, and LT) and fimbriae adhesins [17]. ETEC are a significant and common cause of diarrhoea among suckling and weaned piglets [18]. ETEC colonize the mucosal surface of the small intestine via surface proteins (fimbriae or pilli), producing the following enterotoxins: heat-stable (Sta and STb) and heat-labile (LT) ones, or both [19]. The reported porcine fimbriae are F4 (K88), F5 (K99), F6 (987P), F18, and F41 [20]. Neonatal diarrhoea due to ETEC infection is characterized by high morbidity rates of suckling piglets per litter and is associated with increased economic losses for the swine farms due to an increased mortality rate, reduced growth performance, and increased veterinary cost for treatments and preventive strategies (e.g., antimicrobials, vaccines, alternatives to antibiotics, and biosecurity measures) [21,22]. 

Clostridia are large, rod-shaped bacteria that also form spores that persist in the environment for long periods. *C. perfringens* and *C. difficile* are classified as the main swine enteric clostridial pathogens [23]. *C. perfringens* type C causes severe and foetal necrotic enteritis in neonatal piglets due to the production of α-toxin and β-toxin [23,24,25,26]. Diarrhoea can spread rapidly amongst the herd, inducing high mortality (up to 100%) in affected piglets of non-vaccinated herds [23]. The peracute and acute forms of diarrhoea disease affect piglets mainly at 0–3 days of age [23].

*C. difficile* is a Gram-positive spore-forming bacterium that causes the enteric disease in humans and pigs, among many mammals [27,28,29]. *C. difficile* is widespread in the environment and is a common part of the gastrointestinal microbiota of mammals [30,31]. Previous studies have revealed *C. difficile* to be an emerging pathogen [1], which has been involved in cases of uncontrolled enteritis outbreaks affecting neonatal piglets in the USA and Europe [28,32,33]. Previous studies also reported *C. difficile* strains usually isolated from piglets that have infected humans in North America and Europe [34,35] and play a serious role in human diarrhoea [36].

The fundamental tools to tackling neonatal diarrhoea are a good knowledge of epidemiology, an efficient diagnostic approach, as well as appropriate control or preventive strategies [22]. For example, to control enteric colibacillosis in neonatal piglets, it is crucial to better comprehend the pathotypes and virotypes of *E. coli* and the predisposing factors that allow the bacterium to cause diseases, as well as to apply rapid diagnostic methods and efficient control or preventive tools [22]. For a correct diagnosis of neonatal piglet diarrhoea, detecting the presence of pathogenicity factors is fundamental, as many agents are included in bacterial flora and bacterial isolation is not proof that these agents cause diarrhoea. Multiplex polymerase chain reaction (PCR) is a very effective technique to detect virulence factors, such as the presence of fimbriae F4, F5, F6, and LT of *E. coli*, and the β-toxin of *C. perfringens* type C [23]. Multiplex PCR approaches enable the amplification of several genes simultaneously, reducing the cost and time needed [37,38,39,40]. FTA (Flinders Technology Associates) cards provide important advantages, such as sample storage, transport, and extraction, leading to a decrease in the cost and time needed for molecular diagnosis. FTA cards have been widely used to extract and stabilize DNA from samples of human and swine clinical cases [41,42,43,44,45,46]. The method of FTA cards is reported to be a valid diagnostic tool, and it can be used for a short period (24 h) for the storage and transport of live bacteria, specifically Gram-positive types [44]. 

Multidrug resistance genes among ESBL-producing *E. coli* strains retrieved from feces of pigs were reported, underlining the issue of antimicrobial resistance [47,48,49,50,51]. Various non-antibiotic strategies, including feed additives such as acidifiers or probiotics, have been proposed in swine herd health management strategies as other optional prophylactic or therapeutic protocols [52,53,54,55,56,57,58,59,60,61,62,63,64,65,66,67,68,69,70,71]. Probiotic supplementation in feed is beneficial for improving the immune response and growth performance, maintaining gut health, and controlling enteric infections due to its antimicrobial effects on several enteric pathogens by stimulating the growth of beneficial microorganisms in the gut [54,55,62,63,64,65,66,67,68,69]. Previous studies have reported that dietary acidifiers provide prophylactic effects such as antibiotics do, mainly by limiting the growth of enteric pathogens and simultaneously providing the opportunity for the proliferation of beneficial organisms [56,57,58,59,69,70,71]. 

There are limited published data about the aetiology of neonatal piglet diarrhoea in the Greek swine industry, especially on the prevalence of *C. difficile*. The objective of this study was to investigate the detection of ETEC and *C. difficile* or *C. perfringens* type C pathogens in neonatal piglet diarrhoea, as well as the factors contributing to their prevention in commercial pig farms.

## 2. Materials and Methods

### 2.1. Study Design

#### 2.1.1. Description of Farms, Criteria for Inclusion, and Study Groups

The present study was carried out from January 2020 to October 2021 in Greece. A total of 26 commercial farrow-to-finish pig farms, with an overall population of 12,380 sows comprising approximately 24.5% of the Greek swine industry, representing different regions with high pig density, as well as with different capacities, were included in the study (Table 1). Inclusion criteria for the participated commercial pig farms were the ability to vaccinate sows against *E. coli* (F4ab, F4ac, F5, F6, and LT) and *C. perfringens* type C (beta toxoid) using commercial vaccines (intramuscularly, 2 mL per animal) in a routine program 15–20 days before farrowing. The pig farmers participated voluntarily and provided data about the possible use of acidifiers or/and probiotics in gestation and lactation feed. Moreover, the administration or not of injectable antibiotics to sows on the first day of farrowing was recorded. The injectable antibiotic that was used in sows of selected farms was amoxicillin LA at the dose of 15 mg/kg of body weight (BW) after cultivation and antibiogram in samples of former clinical cases to prevent postpartum dysgalactia syndrome (PDS). Moreover, some of the selected farms used, according to the manufacturer’s guidelines, acidifiers based on commercial products containing a combination of formic acid, propionic acid, phosphoric acid, lactic acid, and acetic acid and/or probiotics based on commercial products with *Bacillus licheniformis* and *Bacillus subtilis* (the used commercial products are analytically presented in Supplementary File S1). The study groups of the trial are presented in Table 2.

#### 2.1.2. Sampling and Laboratory Examinations

**Figure 1:**
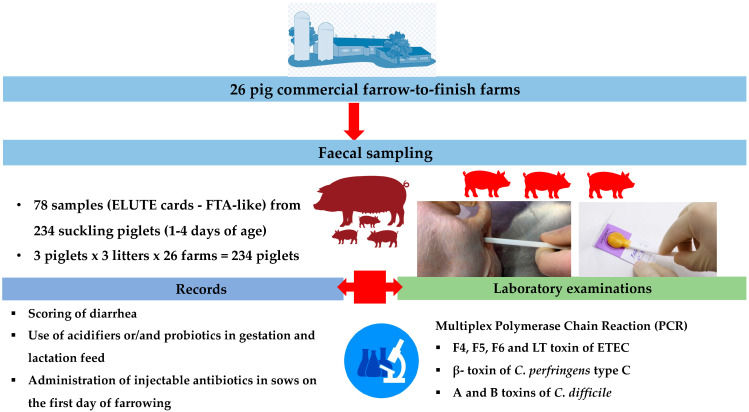
A flowchart of study design (animals, sampling, and laboratory exams).

In total, 78 pooled faecal samples were collected from 234 suckling piglets (1–4 days of age) with diarrhoea (Figure 1). No medication or treatment was given to the selected piglets. Concerning diarrhoea, the sampling time was random. The collected samples were initially screened for the presence of *E. coli* and *C. difficile* or *C. perfringens* via cultivation on MacConkey and anaerobic blood agar, respectively [72]. A modified scoring system created by Pedersen et al. [73] based on faecal consistency was applied for each selected piglet for faecal sampling, as follows: scores 1 (soft faeces), 2 (mild diarrhoea), or 3 (severe diarrhoea). Three rectal swab samples per litter (1 pool) were collected randomly from suckling piglets with symptoms of diarrhoea for three litters per farm (3 piglets × 3 litters × 26 farms = 234 piglets = 78 faecal pool samples). Subsequently, the samples were pooled on ELUTE cards (FTA-like) according to the manufacturer’s instructions (Enterocheck^®^, Hipra, Spain). The DNA extraction process, as well as the one-step multiplex PCR technique to detect genes that codify the adhesion factors F4, F5, F6, and the LT toxin of enterotoxigenic *E. coli* (ETEC), β-toxin of *C. perfringens type C*, and toxins A and B of *C. difficile*, were carried out using specific probes according to laboratory guidelines (Laboratorios Hipra, Amer, Girona, Spain) [74,75,76]. The results were determined as negative (−) based on the Cycle threshold (Ct) values (>38.5 Ct value). Positive samples were categorized into three categories: pos (+): a low quantity of genetic material of the tested pathogens was detected (35–38.5 Ct value); pos (++): a moderate quantity of genetic material of the tested pathogens was detected (30–35 Ct value); pos (+++): a large quantity of genetic material of the tested pathogens was detected (<30 Ct value).

### 2.2. Statistical Analysis

The results were statistically analysed using R programming language [77], and all graphs were created with Seaborn, a Python data visualization library based on matplotlib [78]. The results of the detection of the genes F4, F5, and F6 of ETEC, *E. coli* enterotoxin LT, β-toxin of *C. perfringens*, and toxins A and B of *C. difficile* in faeces of piglets were analysed statistically three times based on the following groups: None, AB, PR, AC, PR + AC, PR + AB, AC + AB, and AB + PR + AC. The results were analysed using the Kruskal–Wallis test [79]. All comparisons were performed at a significant level of *p* < 0.05. Power analysis with G-Power software (version 3.1.) was performed to estimate the study power and the minimum sample size. The actual power of this study was greater than 95% for a total sample size of 78 samples.

## 3. Results

The percentages (proportions) of 26 farms when at least one sample was positive and of 78 samples where ETEC F4, ETEC F5, ETEC F6, enterotoxin LT, and β-toxin of *C. perfringens* type C and toxins A and B of *C. difficile* were detected are presented in Table 3.

**Figure 2:**
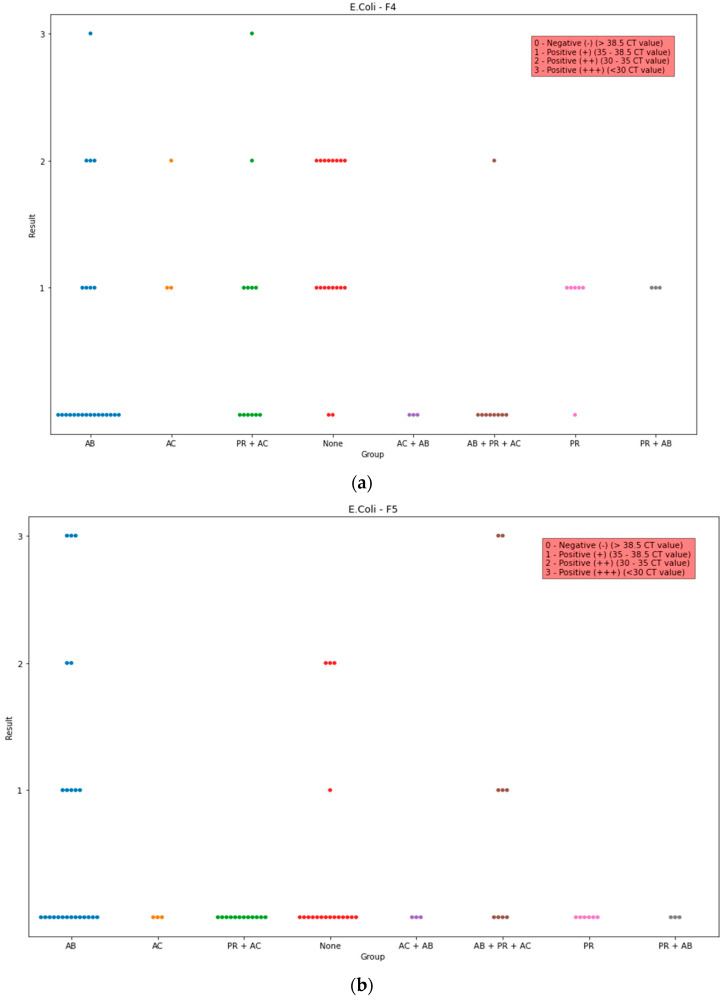

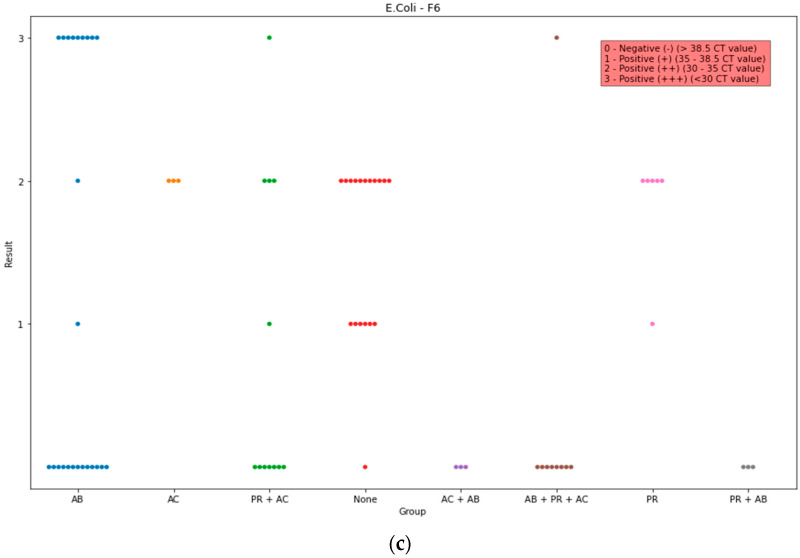
(**a**) Observed data points of the scores of the PCR detected ETEC F4 in all groups. The x-axis indicates the administration group, and y-axis indicates the amount of the detected genes. (**b**) Observed data points of the scores of the PCR detected ETEC F5 in all groups. The x-axis indicates the administration group, and the y-axis indicates the amount of the detected genes. (**c**) Observed data points of the scores of the PCR detected ETEC F6 in all groups. The x-axis indicates the administration group, and the y-axis indicates the amount of the detected genes.

Regarding the detection of F4 gen, the quantity was significantly higher in group None in comparison to those in groups AB (*p* = 0.018), AB + PR + AC (*p* = 0.014), AC + AB (*p* = 0.0117), and PR + AC (*p* = 0.04). A statistical difference was found between group AB + PR + AC in comparison and groups AC (*p* = 0.02), PR (*p* = 0.02), and PR + AB (*p* = 0.02). Lastly, groups AC + AB and PR were found to be statistically different (*p* = 0.03). Groups AB, AB + PR + AC, AC + AB had the lowest median (higher quantity of genetic material), while the highest median (lower quantity of genetic material) was observed in groups AC, PR, PR + AB, and None (Figure 2a). 

Statistical analysis of ETEC F5 detection in the faeces of piglets showed that the proportion was significantly lower in group PR in comparison to that in group AB + PR + AC (*p* = 0.04), and it was lower in group PR + AC compared to those in groups AB (*p* = 0.01) and AB + PR + AC (*p* = 0.004). Group AB + PR + AC had the highest median (Figure 2b). 

Regarding the detection of ETEC F6 in the faeces of piglets, the proportion was significantly lower in group PR + AB in comparison to those in groups AC (*p* = 0.04), None (*p* = 0.005), and PR (*p* = 0.01). Similarly, group AB + PR + AC had a significant difference in comparison to groups PR (*p* = 0.001) and PR (*p* = 0.007). Lastly, there was a statistical difference between groups AC + AB and PR (*p* = 0.001). The highest median was in groups AC, PR, and None (Figure 2c). 

**Figure 3:**
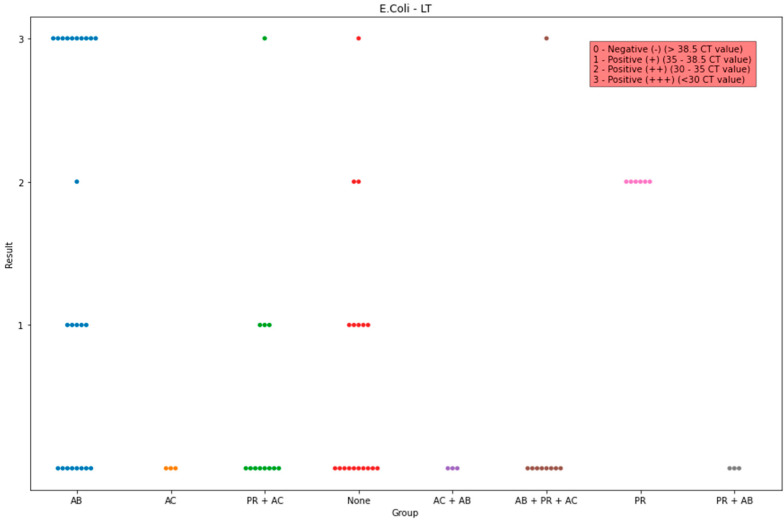
Observed data points of the scores of the PCR detected *E. coli* toxin LT in all groups. The x-axis indicates the administration group, and the y-axis indicates the amount of the detected genes.

Statistical analysis of the detection of enterotoxin LT showed that the proportion was significantly higher in group PR in comparison to those in groups AC (*p* = 0.007), AC + AB (*p* = 0.007), and None (*p* = 0.003). Statistical differences were also found between groups AB and B + PR + AC (*p* = 0.01), AB and None (*p* = 0.03), PR and PR + AB (*p* = 0.007), and PR + AC and AB (*p* = 0.02) and PR (*p* = 0.003). The highest medians were in groups PR and AB (Figure 3).

**Figure 4:**
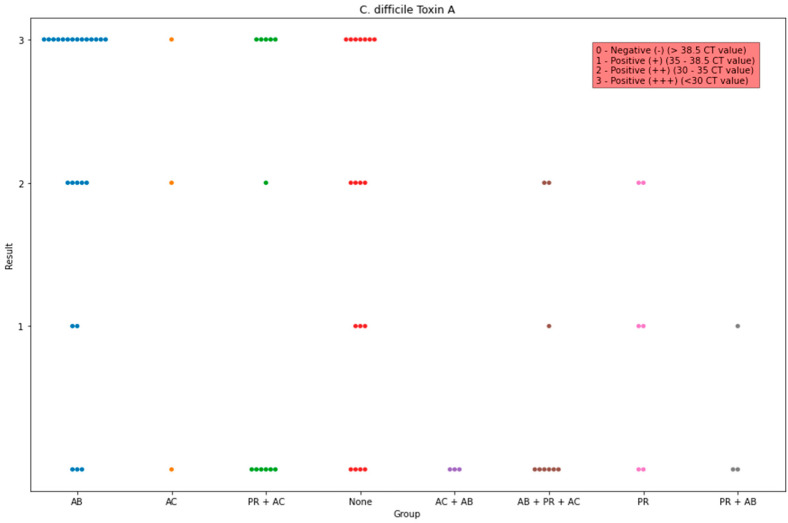
Observed data points of the scores of the PCR detected toxin A of *C. difficile* in all groups. The x-axis indicates the administration group, and the y-axis indicates the amount of the detected genes.

Concerning the detection of toxin A of *C. difficile*, the proportion was found significantly higher in group AB in comparison to those in groups AB + PR + AC (*p* < 0.001), AC + AB (*p* = 0.009), PR (*p* = 0.01), and PR + AB (*p* = 0.01), and it was lower in group AB + PR + AC in comparison to group None (*p* = 0.01). Group AB had the highest median, while groups AB + PR + AC, AC + AB, and PR + AB had the lowest median (Figure 4).

**Figure 5:**
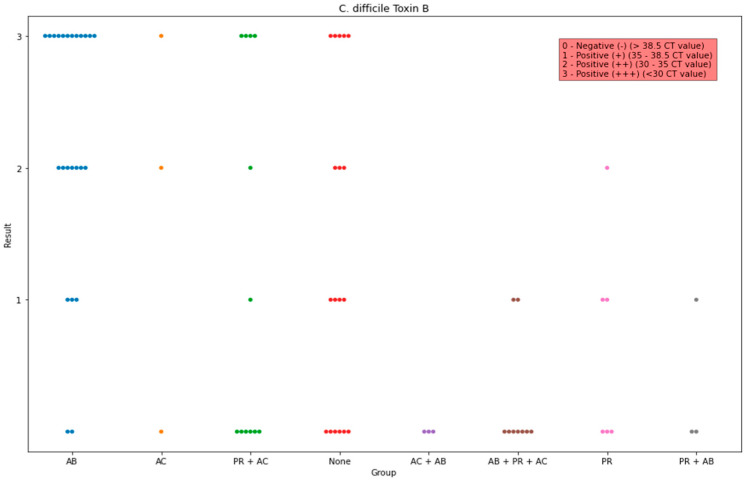
Observed data points of the scores of the PCR detected toxin B of *C. difficile* in all groups. The x-axis indicates the administration group, and the y-axis indicates the amount of the detected genes.

Moreover, statistical analysis of the detected toxin B of *C. difficile* showed that the proportion is significantly higher in group AB in comparison to those in groups AB + PR + AC (*p* < 0.001), AC + AB (*p* = 0.0078, None (*p* = 0.03), PR (*p* = 0.003), and PR + AB (*p* = 0.01). Lastly, a significant difference between groups AB + PR + AC and None (*p* = 0.165) was found. For the detection of toxin B of *C. difficile*, the lowest median was in groups AC + AB, None, and AB + PR + AC (Figure 5).

**Figure 6:**
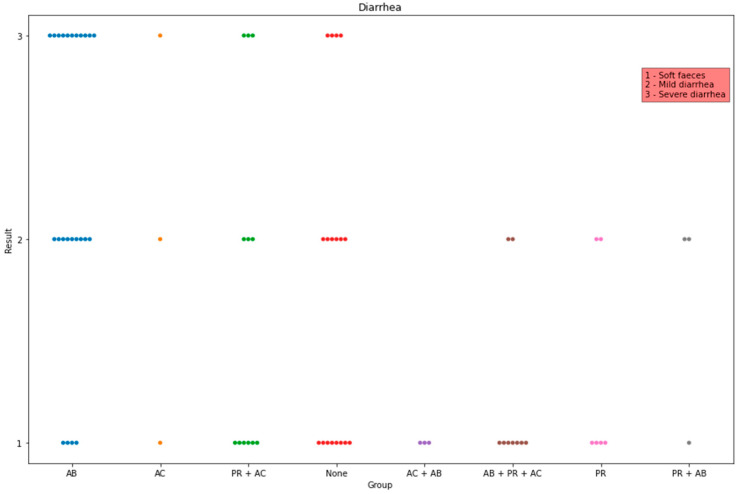
Observed data points of diarrhoea scores in all groups. The x-axis indicates the administration group, and the y-axis indicates the amount of the detected scores.

Concerning the score of piglet diarrhoea, it was found significantly higher in group AB in comparison to those in groups AB + PR + AC (*p* = 0.001), AC + AB (*p* = 0.015), None (*p* = 0.04), and PR (*p* = 0.01). The highest median was in group AB, while the lowest one was in groups AB + PR + AC, AC + AB, and PR (Figure 6).

## 4. Discussion

Diarrhoea in neonatal piglets up to 4 days of age is a common issue in many sow herds worldwide, and its diagnosis remains an ongoing challenge for swine practitioners. Among the causal agents of neonatal diarrhoea, *E. coli* is present in swine herds around the world [1,13]. The results of our study confirmed that *E. coli* is an enteric pathogen of concern for Greek swine farms, even if vaccinations against *E. coli* are given to sows. However, in contrast to previous studies, we noticed a higher prevalence of F4 and F6, as well as LT toxins, in comparison to that of F5 [80]. Based on our results, 69.23% (18/26) of those in the farms were F4-positive, 30.77% (8/26) were F5-positive, 61.54% (16/26) were F6-positive, 42.31% (11/26) were F4- and enterotoxin LT-positive, 19.23% (5/26) were F5- and LT-positive, 42.31% (11/26) were F6- and LT-positive, and in 57.69% (15/26) of those in the farms, *E. coli* enterotoxin LT was found. However, our study did not investigate heat-stable (Sta and STb) *E. coli* enterotoxins due to the absence of this option in the ELUTE cards used according to the manufacturer’s instructions (Enterocheck^®^, Hipra, Madrid, Spain) [74,75,76].

In recent years, the focus of vaccination strategies against neonatal and post-weaning diarrhoea has been on anti-adhesin strategies, as this is the initial step of ETEC pathogenesis. However, while these vaccines have provided some protection against ETEC infections, there is still no universally effective ETEC vaccine that is commercially available [81]. A comprehensive approach that includes an appropriate vaccination program for the sow herd, the adequate intake of piglet colostrum and milk, and effective preventive strategies could help reduce the incidence of infectious diarrhoea in piglets. Several risk factors associated with clinical forms of neonatal diarrhoea have been reported to enhance protection against enteric pathogens, including passive immunity transferred by colostrum and milk [82,83], environmental conditions (e.g., temperature and humidity) [21,84], herd health management [85], the inadequate intake of colostrum, parity of the sows, and infection pressure by specific pathogens among the herd [85,86,87].

Various studies suggest that further investigations of *C. difficile* as an etiological agent in neonatal diarrhoea are needed [1,88,89]. Previous studies reported that *C. difficile* could be an etiological agent of enteritis in neonatal piglets [90,91,92]. While it is not considered to be a primary diarrheic pathogen in pigs based on epidemiological studies [93,94,95,96], other studies indicate that *C. difficile* is an emerging pathogen in neonatal diarrhoea [1,97,98]. These findings highlight the need for farmers to use recently developed commercial vaccines that provide immunization against *C. difficile* to sows to prevent neonatal diarrhoea via passive immunity. A new commercial vaccine for the immunization of sows with *C. difficile* (toxins A and B) and *C. perfringens* type A was recently registered in Europe, resulting in a significant reduction in diarrhoea and productive losses caused by *C. difficile* and *C. perfringens* type A [99].

While a previous study reported that pathogenic *E. coli* was only found in combination with other pathogens in Spanish swine farms [82], our study found that *E. coli* is present in combination with *C. difficile*, but no detection of *C. perfringens* type C was noticed among the 26 pig farms studied. Even if *C. difficile* causes disease in piglets worldwide, it is considered to be much less important than other enteric pathogens are from a global perspective [100]. For example, a low prevalence of *C. perfringens* type C (1,4%) in herds was reported in Poland [101]. Our results regarding the absence of *C. perfringens* type C in faecal samples from newborn piglets with diarrhoea are consistent with those in previous studies, which included a larger number of herds and samples [3]. These findings may be explained by the sufficient protection of routine vaccinations for sows against *C. perfringens* type C [1,3]. However, in contrast to our results, these studies did not find a relationship between *C. difficile* and diarrhoeal status. 

Our study found that administering antibiotics alone to sows did not reduce the detection of ETEC antigens. However, combining antibiotics with probiotics or acidifiers or both resulted in better outcomes. This is supported by several studies that have reported the beneficial effects of probiotics as a feed additive in the health and performance of piglets and sows [102,103,104]. The supplementation of probiotics in the feed of gestating and lactating sows has been shown to improve their health status and reproductive performance, as well as increase the production of immunoglobulins in colostrum and milk, resulting in a decrease in the incidence of neonatal diarrhoea [105,106,107,108,109]. Additionally, the administration of acidifiers in pig diets can enhance their growth performance and modulate the intestinal microbiota, reducing gastric pH and delaying the multiplication of enterotoxigenic *E. coli* [110,111]. The supplementation of acidifiers in sow feed could influence the mother’s microbiota and affects those of piglets [112]. Our results could support the findings of previous trials relating to the administration of acidifiers in sow gestation feed, which reported beneficial effects on the sows’ performance during lactation [113,114] and their gut microbiome, reducing the *E. coli* counts during farrowing and weaning [115].

Feed additives, including probiotics and acidifiers, have been explored as antibiotic alternatives, but their effectiveness varies [116]. Our study found that administering antibiotics alone did not reduce the detection of pathogen toxins (such as enterotoxin LT and toxin A of *C. difficile*), but combining antibiotics with probiotics or acidifiers decreased the toxin levels. Feeding sows probiotics or acidifiers also resulted in lower neonatal piglet diarrhoea scores compared to those who received nothing or only injectable antibiotics. In contrast to our results, Greeff et al. [117] reported that oral amoxicillin administration during the last week of gestation to sows can modulate the gut development of their piglets for a period of at least 5 weeks after the last antibiotic administration.

It is worth noting that in the case of *C. difficile*, the highest median toxin detection was found in the group that received antibiotics alone, while a lower detection rate was observed in groups that received alternatives to antibiotics, such as probiotics and acidifiers, either alone or in combination with antibiotics. In humans, *C. difficile* infection (CDI) is becoming increasingly difficult to treat due to severe antibiotic resistance, and for this reason, there are very limited treatment options [118,119]. Currently, only three antibiotics (metronidazole, vancomycin, and fidaxomicin) are available for CDI treatment [120]. These antibiotics are forbidden for use in swine. Fry et al. [121] reported that significant proportions of *C. difficile* in swine are toxigenic and are often associated with antimicrobial resistance genes, although they are not resistant to drugs that are used to treat CDI. A connection between the presence of *C. difficile* and diarrhoea or antibiotic treatments in piglets has not been proven thus far [122,123,124]. Moreover, a previous study by Schneeberg et al. [125] reported that emerging human-pathogenic *C. difficile* PCR ribotypes were the predominant PCR ribotypes in piglets in Germany. However, they did not find a link between the presence of *C. difficile* in piglets and antibiotic treatment or diarrhoea in piglets. 

Our findings highlight the potential benefits of using alternatives to antibiotics, such as probiotics and acidifiers, in swine feed to improve the health and performance of both sows and their piglets. In addition, our results suggest that further research is needed to investigate the potential use of these alternatives in the treatment of CDI in swine and humans, either alone or in combination with antibiotics.

## 5. Conclusions

In our research study, despite routine vaccinations of sows against *E. coli*, a high prevalence of positive samples was found, revealing that *E. coli* remains a main pathogen of great importance in neonatal pig diarrhoea. Additionally, our study reports, for the first time in Greece, the involvement of *C. difficile* in most clinical cases of neonatal pig diarrhoea in commercial pig farms. Therefore, the need for herd immunization against *C. difficile* may be necessary. However, these findings reveal the necessity of extended future studies on the epidemiology of *C. difficile* in pigs and the need for research on this pathogen in the future, as well as the need for herd immunization against *C. difficile*. Maternal interventions with antibiotics in combination with probiotics or acidifiers appear to have beneficial effects in terms of neonatal pig diarrhoea, reducing the detection of ETEC antigens and the enterotoxin LT of *E. coli*.

## Figures and Tables

**Table 1:** Overview of the 26 commercial pig farms included in the present study: number per geographic region, the capacity of sows, and the number of samples.

Region of Greece	Number of Farms	Capacity of Sows per Farm				Number of Faecal Pool Samples
		<100	101–300	301–500	>500	
Central Greece	10	2	3	1	4	30
North Greece	7	0	4	1	2	21
West Greece	5	0	2	0	3	15
South Greece	4	1	1	1	1	12
Total	26	3	10	3	10	78

**Table 2:** Overview of the study groups according to the use of antibiotics, probiotics, or acidifiers.

Groups of the Trial Farms Based on Their Routine Program in Sows							
Non-Use of AB *, PR **, and AC ***	Injectable AB at 1st Day of Farrowing	Use in Pre-Farrowing Feed			Combination of Injectable AB and PR or/and AC in Pre-Farrowing Feed		
Group None	Group AB	Group PR	Group AC	Group PR + AC	Group PR + AB	Group AC + AB	Group AB + PR + AC
6	8	2	1	4	1	1	3

* AB: antibiotics. ** PR: probiotics. *** AC: acidifiers.

**Table 3:** Percentage (%) and number (no) of positive pig farms (when at least one sample was positive) and positive samples in the detection, by PCR of ETEC F4, F5, and F6 genes, enterotoxin LT, β toxin of *C. perfringens*, and toxins A and B of *C. difficile*.

Analytical Target	% Positive Farms (No Farms)	% Positive Samples (No Pool Samples)
F4 gene	69.23% (18)	53.85% (42)
F5 gene	30.77% (8)	24.36% (19)
F6 gene	61.54% (16)	55.13% (43)
Enterotoxin LT	57.69% (15)	44.88% (35)
F4 gene + Enterotoxin LT	42.31% (11)	25.64% (20)
F5 gene + Enterotoxin LT	19.23% (5)	16.67% (13)
F6 gene + Enterotoxin LT	42.31% (11)	32.05% (25)
β-toxin—*C. perfringens* type C	0.00% (0)	0.00% (0)
Toxin A—*C. difficile*	84.62% (22)	65.38% (51)
Toxin B—*C. difficile*	88.46% (23)	61.54% (48)

## Data Availability

Not applicable.

## Supplementary Materials

The following supporting information can be downloaded at: https://www.mdpi.com/article/10.3390/life13051092/s1, Supplementary File S1: used commercial products in farms.
