# Supplementary material for: Detection of Enterotoxigenic Escherichia coli and Clostridia in the Aetiology of Neonatal Piglet Diarrhoea: Important Factors for Their Prevention
Source: Life (Basel). 2023 Apr 27;13(5):1092. doi: 10.3390/life13051092 (PMC10223568; doi:10.3390/life13051092)
Supplement: Supplementary file 1 [file life-13-01092-s001.zip › life-2341765-supplementary.pdf]

Farm no	Group	Commercial products	Composition / Dose
Farm 1	Group None	-	
Farm 2	Group AB	Citramox® LA (Laboratorios Karizoo S.A.)	Amoxicillin 150.00 mg/ml (15 mg of amoxicillin/kg BW)
Farm 3	Group AB	Vetrimoxin® LA (Ceva Animal Health)	Amoxicillin 150.00 mg/ml (15 mg of amoxicillin/kg BW)
Farm 4	Group None	-	
Farm 5	Group AC	Acid LAC® XT dry (Kemin)	Lactic acid, fumaric acid, citric acid and formic acid (4 Kg/t feed)
Farm 6	Group None	-	
Farm 7	Group AB	Betamox® LA (Norbrook Laboratories)	Amoxicillin 150.00 mg/ml (15 mg of amoxicillin/kg BW)
Farm 8	Group AC +AB	Vetrimoxin® LA (Ceva Animal Health)<br>+<br>Biotronic® (BIOMIN GmbH, DSM)	Organic acid blend (formic, propionic and acetic acid), cinnamaldehyde and a permeabilising substance (3 kg/t feed)<br>+<br>Amoxicillin 150.00 mg/ml (15 mg of amoxicillin/kg BW)
Farm 9	Group PR + AC	Bioplus YC (Biochem)<br>+<br>Arma (O3 Benelux GmbH)	*Bacillus licheniformis* (DSM 5749) and *Bacillus subtilis* (DSM 5750) in a 1:1 ratio (1.6 x 10^9 CFU + 1.6 x 109 CFU per g product, 400 g/t feed)<br>+<br>Formic acid, propionic acid, phosphoric acid, lactic acid, and acetic acid (3 Kg/t feed)
Farm 10	Group PR + AC	Bioplus YC (Biochem)<br>+<br>Arma (O3 Benelux GmbH)	*Bacillus licheniformis* (DSM 5749) and *Bacillus subtilis* (DSM 5750) in a 1:1 ratio (1.6 x 10^9 CFU + 1.6 x 109 CFU per g product, 400 g/t feed)<br>+<br>Formic acid, propionic acid, phosphoric acid, lactic acid, and acetic acid (3 Kg/t feed)
Farm 11	Group None	-	
Farm 12	Group AB + PR + AC	Vetrimoxin® LA (Ceva Animal Health)<br>+<br>Bioplus YC (Biochem)<br>+	Amoxicillin 150.00 mg/ml (15 mg of amoxicillin per kg BW)<br>+

		Acid LAC® XT dry (Kemin)	*Bacillus licheniformis* (DSM 5749) and *Bacillus subtilis* (DSM 5750) in a 1:1 ratio (1.6 x 10^9 CFU + 1.6 x 109 CFU per g product, 400 g/t feed)<br>+<br>Formic acid, propionic acid, phosphoric acid, lactic acid, and acetic acid (3 Kg/t feed)
Farm 13	Group AB	Vetrimoxin ® LA (Ceva Animal Health)	Amoxicillin 150.00 mg/ml (15 mg of amoxicillin/kg BW)
Farm 14	Group PR + AB	Citramox® LA (Laboratorios Karizoo S.A.)<br>+<br>Bioplus YC (Biochem)	*Bacillus licheniformis* (DSM 5749) and *Bacillus subtilis* (DSM 5750) in a 1:1 ratio (1.6 x 10^9 CFU + 1.6 x 109 CFU per g product, 400 g/t feed)<br>+<br>Amoxicillin 150.00 mg/ml (15 mg of amoxicillin/kg BW)
Farm 15	Group AB	Citramox® LA (Laboratorios Karizoo S.A.)	Amoxicillin 150.00 mg/ml (15 mg of amoxicillin/kg BW)
Farm 16	Group PR + AC	Bioplus YC (Biochem)<br>+<br>Acid LAC® XT dry (Kemin)	*Bacillus licheniformis* (DSM 5749) and *Bacillus subtilis* (DSM 5750) in a 1:1 ratio (1.6 x 10^9 CFU + 1.6 x 109 CFU per g product, 400 g/t feed)<br>+<br>Lactic acid, fumaric acid, citric acid and formic acid (4 Kg/t feed)
Farm 17	Group AB + PR + AC	Betamox® LA (Norbrook Laboratories)<br>+<br>BioPlus® YC (Biochem)<br>+<br>Arma (O3 Benelux GmbH)	Amoxicillin 150.00 mg/ml (15 mg of amoxicillin/kg BW)<br>+<br>*Bacillus licheniformis* (DSM 5749) and *Bacillus subtilis* (DSM 5750) in a 1:1 ratio (1.6 x 10^9 CFU + 1.6 x 109 CFU per g product, 400 g/t feed)<br>+<br>Formic acid, propionic acid, phosphoric acid, lactic acid, and acetic acid (3 Kg/t feed)
Farm 18	Group AB	Betamox® LA (Norbrook Laboratories)	Amoxicillin 150.00 mg/ml (15 mg of amoxicillin/kg BW)
Farm 19	Group AB	Betamox® LA (Norbrook Laboratories)	Amoxicillin 150.00 mg/ml (15 mg of amoxicillin/kg BW)
Farm 20	Group None	-	

Farm 21	Group PR	BioPlus® YC (Biochem)	*Bacillus licheniformis* (DSM 5749) and *Bacillus subtilis* (DSM 5750) in a 1:1 ratio (1.6 x 10^9 CFU + 1.6 x 109 CFU per g product, 400 g/t feed)
Farm 22	Group PR + AC	BioPlus® YC (Biochem)<br>+<br>Acid LAC® XT dry (Kemin)	*Bacillus licheniformis* (DSM 5749) and *Bacillus subtilis* (DSM 5750) in a 1:1 ratio (1.6 x 10^9 CFU + 1.6 x 109 CFU per g product, 400 g/t feed)<br>+<br>Lactic acid, fumaric acid, citric acid and formic acid (4 Kg/t feed)
Farm 23	Group AB	Betamox® LA (Norbrook Laboratories)	Amoxicillin 150.00 mg/ml (15 mg of amoxicillin/kg BW)
Farm 24	Group AB + PR + AC	Citramox® LA (Laboratorios Karizoo S.A.)<br>+<br>Bioplus YC (Biochem)<br>+<br>Acid LAC® XT dry (Kemin)	Amoxicillin 150.00 mg/ml (15 mg of amoxicillin/kg BW)<br>+<br>*Bacillus licheniformis* (DSM 5749) and *Bacillus subtilis* (DSM 5750) in a 1:1 ratio (1.6 x 10^9 CFU + 1.6 x 109 CFU per g product, 400 g/t feed)<br>+<br>Lactic acid, fumaric acid, citric acid and formic acid (4 Kg/t feed)
Farm 25	Group None	-	
Farm 26	Group PR	Bioplus YC (Biochem)	*Bacillus licheniformis* (DSM 5749) and *Bacillus subtilis* (DSM 5750) in a 1:1 ratio (1.6 x 10^9 CFU + 1.6 x 109 CFU per g product, 400 g/t feed)
